# The Prognostic Value of Circulating Soluble Programmed Death Ligand-1 in Cancers: A Meta-Analysis

**DOI:** 10.3389/fonc.2020.626932

**Published:** 2021-02-25

**Authors:** Pei Huang, Wei Hu, Ying Zhu, Yushen Wu, Huapeng Lin

**Affiliations:** ^1^ Department of Intensive Care Unit, Affiliated Hangzhou First People’s Hospital, Zhejiang University School of Medicine, Hangzhou, China; ^2^ Department of Oncology, The First Affiliated Hospital of Chongqing Medical University, Chongqing, China; ^3^ Chongqing Key Laboratory of Molecular Oncology and Epigenetics, The First Affiliated Hospital of Chongqing Medical University, Chongqing, China

**Keywords:** soluble programmed death ligand 1, cancers, prognosis, survival, meta-analysis

## Abstract

**Background:**

Studies on the prognostic value of the soluble programmed death ligand 1 (sPD-L1) in cancer patients have not yielded consistent results.

**Objective:**

This meta-analysis was performed to assess the association between sPD-L1 and the prognosis of cancer patients.

**Methods:**

Published articles in Pubmed, EMBASE, and Cochrane clinical trial databases were searched from the inception to September 2020. Overall survival (OS), progression-free survival (PFS), recurrence-free survival (RFS), and disease-free survival (DFS) data were evaluated using a hazard ratio (HR) at 95% confidence interval (95% CI).

**Results:**

A total 31 studies involving 17 tumors and 3,780 patients were included. The overexpression of sPD-L1 was associated with shorter OS (HR 1.85, 95% CI 1.59–2.15, I^2^ = 33%). High sPD-L1 had worse PFS (HR 2.40, 95% CI 1.55–3.72, I^2^ = 83%), and worse DFS (HR 2.92, 95% CI 2.02–4.29, I^2^ = 40%), without significant statistical difference in RFS (HR 2.08, 95% CI 0.99–4.40, I^2^ = 0%).

**Conclusions:**

High sPD-L1 levels were associated with worse survival prognosis in cancer patients. The sPD-L1 may be a potential prognostic, non-invasive, and dynamic monitoring biomarker for cancers in the future.

## Introduction

Immune checkpoint inhibitors such as anti-PD-1/PD-L1 have remarkable clinical benefits in a variety of tumors (1, 2). The expression of programmed death ligand 1 (PD-L1) is often observed in a variety of cancers. The combination of PD-L1 and its receptor PD-1 on activated T cells suppresses antitumor immunity by counteracting T cell activation signals. Antibody-based PD-1/PD-L1 inhibitors can induce durable tumor remission of various advanced cancer types.

PD-L1 exists in two forms; membrane-bound and soluble form (3). Studies have shown that soluble PD-L1 (sPD-L1) may be derived from the shedding of tumor cells or the release of immune cells in tumor tissues (3, 4). Evidence shows that the surfaces of exosomes secreted by tumor cells have biologically active PD-L1, which can suppress immune responses (5). The circulatory cell expressing PD-L1 still retains its biological activity, and it can specifically bind the PD-1 receptor of the T cells in peripheral blood, thereby activating the PD-1/PD-L1 pathway and establishing systemic immunosuppression (6, 7). Moreover, sPD-L1 is a prognostic marker of tumor treatment (8).

Studies on the relationship between sPD-L1 and the prognosis of cancer patients have yielded contrasting results. In NSCLC, studies by He et al. showed that patients with high sPD-L1 have a reduced risk of death (9). However, data from Okuma et al. showed that lung cancer patients with high sPD-L1 have an increased risk of death (10). In gastric cancer and nasopharyngeal carcinoma, there is no correlation between soluble PD-L1 levels and overall survival (4, 11). This systematic review and meta-analysis aimed at evaluating the value and clinical significance of sPD-L1 in the survival prognosis of cancer patients.

## Materials and Methods

### Search Strategy and Inclusion Criteria

Relevant articles published in various databases were searched since the inception of the databases to September 2020. The Pubmed, EMBASE, and Cochrane clinical trial databases were searched for the meta-analysis. The Clinicaltrial.gov, American Society of Clinical Oncology (ASCO) and ESMO databases were also searched. Search terms were composed of various combinations of “soluble PD-L1”, “sPD-L1”, “serum PD-L1”, “plasma PD-L1”, “circulatory PD-L1”, “blood PD-L1”, “programmed death ligand 1”, “B7-H1” and “cancer”, “neoplasm”, “carcinoma”, “lymphoma”, “sarcomas”. The language of the article was not restricted.

The inclusion criteria for various studies were: i. The included patients were all pathologically diagnosed tumor patients; ii. With analyzable soluble PD-L1 data; iii. With sufficient clinical features and data that could be combined to analyze soluble PD- L1 and survival prognosis; iv. Had their sPD-L1 levels detected by enzyme-linked immunosorbent assay (ELISA). The exclusion criteria were: i. Case reports, reviews, and tissue immunohistochemical (IHC) detection for PD-L1; ii. Animal experiments; iii. No direct analysis of the relationship between soluble PD-L1 and survival prognosis; iv. Incomplete data or non-original research.

### Data Extraction and Quality Assessment

First, the outcomes of each study were extracted, including overall survival (OS), progression-free survival (PFS), recurrence-free survival (RFS), and disease-free survival (DFS) prognosis data. Next, the baseline data of the studies were extracted and analyzed; publication years, study type, patient characteristics, treatment methods, and the cut-off value of sPD-L1, etc. Based on the purpose of our research, OS was the primary outcome of interest. All data screening and extraction processes were independently performed by two investigators.

The Newcastle-Ottawa Quality Assessment Scale (NOS) was used to assess the quality of the study. The NOS consists of three parts: selection (4 points), comparability (2 points), and outcome evaluation (3 points). **Table 1** shows the NOS scores of the included studies. All processes were independently performed by two reviewers and in case of disagreements, they were discussed to reach a consensus. Studies with an NOS score ≥6 were considered high-quality.

**Table 1:** Characteristics of included studies.

Study/year	Cancer Type	Country/region	Study Type	Included Period	No of Samples	Median Age	Primary Outcome	
Lu et al., 2020 (11)	Nasopharyngeal Carcinoma	China	R	2012–2015	219	48	OS/DFS/RFS	
Ha et al., 2016 (12)	Cholangiocarcinoma	Korea	R	2004–2009	158	59.6	OS	
Meyo et al., 2020 (13)	Lung carcinoma	France	R	2015–2018	51	66	OS/PFS	
Jin et al., 2018 (14)	SCLC	China	R	2010–2016	250	64.5	OS	
He et al., 2020 (9)	NSCLC	China	R	2008–2009	88	59	OS	
Zhao et al., 2017 (15)	NSCLC	China	R	2009–2013	126	65	OS	
Okuma et al., 2017 (10)	Lung carcinoma	Japan	P	2014–2016	96	68.5	OS	
Okuma et al., 2018 (16)	NSCLC	Japan	P	2016–2017	39	69	OS/PFS	
Bai et al., 2018 (17)	NSCLC	Netherlands	R	After 2012	102	61.5	OS/PFS	
Lee 2018 (18)	Hepatocellular carcinoma	Korea	R	NA	78	NA	OS/RFS	
Ma 2019 (19)	Hepatocellular carcinoma	China	NA	2012–2013	114	NA	OS/RFS	
Chang 2019 (20)	Hepatocellular carcinoma	China	R	2008–2014	120	NA	OS/DFS/RFS	
Finkelmeier et al., 2016 (21)	Hepatocellular carcinoma	Germany	P	2009–2015	215	64	OS	
Aghajani et al., 2019 (22)	Thyroid carcinoma	Australia	R	2012–2017	101	47	DFS	
Chiarucci et al., 2020 (23)	Mesothelioma	Italy	R	NA	40	66	OS	
Omura et al., 2020 (24)	Colorectal carcinoma	Japan	R	2013–2015	131	69	OS/DFS	
Tominaga et al., 2019 (25)	Rectal carcinoma	Japan	P	2013–2017	117	61	DFS	
Wang et al., 2016 (26)	NK/T cell lymphoma	China	P	2008–2015	97	42	OS/PFS	
Nagato et al., 2017 (27)	NK/T cell lymphoma	Japan	R	2000–2014	17	52	OS	
Shen et al., 2019 (28)	Peripheral T-cell lymphoma	China	P	2016–2018	80	46.5	OS/PFS	
Rossille et al., 2014 (29)	Large B-Cell lymphoma	France	R	2005–2010	283	NA	OS	
Rossille 2017 (30)	Large B-Cell lymphoma	American	P	NA	225	NA	OS	
Guo et al., 2018 (31)	Hodgkin lymphoma	China	R	2005–2015	108	34.6	OS/PFS	
Buderath et al., 2019 (32)	Ovarian carcinoma	Germany	R	2007–2014	83	68	OS/PFS	
Asanuma et al., 2020 (33)	Soft tissue sarcomas	Japan	R	2009–2016	135	63.4	OS/PFS/RFS	
Frigola et al., 2011 (7)	Renal cell carcinoma	American	R	2003–2007	172	52	OS	
Cheng et al., 2019 (34)	Esophageal carcinoma	China	P	NA	161	NA	OS	
Shigemori 2019 (35)	Gastric carcinoma	Japan	R	2008–2014	180	70	OS/DFS	
Takahashi et al., 2016 (4)	Gastric carcinoma	Japan	R	2011–2015	75	67	OS/PFS	
Bian et al., 2019 (36)	Pancreatic adenocarcinoma	France	R	2012–2016	59	68	OS	
Park 2019 (37)	Pancreatic adenocarcinoma	Korea	P	2013–2015	60	NA	OS/PFS	
Follow-up (Months)	Cut-off	Cut-off Selection	Surgery	Immunotherapy	Chemotherapy	Stage/T stage	NOS score	Conference summary
50	93.7 pg/ml	X-Tile	No surgery	No	NA	I–IV	8	No
95.3	0.94 ng/ml	Minimum P value approach	No surgery	No	NA	Advanced	8	No
26.8	0.156 ng/ml	Lower limit of quantification	No surgery	Nivolumab	Yes	Advanced	7	No
NA	7.1 ng/ml	ROC	No surgery	No	Yes	Advanced	8	No
67	NA	NA	Surgery	No	No	Ia–IIIb	7	No
25	96.5 pg/ml	ROC	No surgery	No	NA	IIIb	8	No
NA	7.32 ng/ml	ROC	No surgery	NA	NA	IIIb–IV	7	No
NA	3.35 ng/ml	ROC	No surgery	Nivolumab	Yes	IV	6	No
NA	NA	NA	NA	No	NA	Advanced	NA	Yes
16.1	19.2 pg/ml	NA	Surgery	No	NA	NA	NA	Yes
NA	NA	NA	NA	No	NA	NA	NA	Yes
NA	11.2 µg/ml	X-Tile	Surgery	No	NA	NA	8	No
9.93	0.8 ng/ml	Median value	Mixed	No	NA	NA	8	No
16	0.44 ng/ml	ROC	Surgery	No	No	I–IVb	8	No
NA	0.07 ng/ml	ROC	No surgery	Multiple therapy*	No	III–IV	6	No
NA	0.08 ng/ml	ROC	Surgery	No	NA	I–III	8	No
33.7	0.16 ng/ml	Median value	Surgery	No	Yes	II–III	8	No
38	3.23 ng/ml	ROC	No surgery	No	Yes	I–II	7	No
90	850 pg/ml	ROC	No surgery	No	Yes	I–IV	6	No
20	176.30 pg/ml	ROC	No surgery	No	Yes	I–IV	7	No
41.4	1.52 ng/ml	MaxStat test	No surgery	No	Yes	III–IV	8	No
NA	1652 pg/ml	Median value	No surgery	No	Yes	I–IV	8	No
47	25.16 ng/ml	ROC	No surgery	No	Yes	I–IV	8	No
NA	6.4 pg/ml	ROC	Surgery	No	NA	II–IV	7	No
42.9	44.26 pg/ml	ROC	Surgery	No	NA	I–III	7	No
45.6	NA	NA	Surgery	No	NA	I–IV	8	No
NA	NA	Median Value	Surgery	No	Yes	Advanced	NA	Yes
NA	0.507 ng/ml	ROC	Surgery	No	NA	I–IV	8	No
NA	1.081 ng/ml	ROC	No surgery	No	Yes	IV	7	No
NA	8.6 ng/ml	ROC	Mixed	No	Yes	Tx-T4	7	No
11.4	4.6 ng/ml	ROC	No surgery	No	Yes	Advanced	6	No

P, prospctive; R, retrospctive; OS, overal survival; PFS, progression-free survival; RFS, recurrence-free survival; DFS, disease-free survival; SCLC, small cell lung cancer; NSCLC, non-small cell lung cancer; ROC, receiver operating characteristic curve; NOS, Newcastle–Ottawa quality assessment scale; *: tezolizumab/durvalumab/ipilimumab/tremelimumab/pembrolizumab/nivolumab.

### Statistical Analysis

The 95% confidence interval (CI) of all results was calculated. All outcomes were directly extracted from the studies. Time-to-event endpoints (OS, PFS, DFS, and RFS) were pooled with the use of hazard ratio (HR). Heterogeneity was assessed using the χ2-based Q test and I^2^ statistics. I^2^ >75% indicated considerable heterogeneity (38). The random-effects model was applied for pooled analysis. The RevMan software (version 5.3) from Cochrane library was used for meta-analysis. Publication bias was evaluated by funnel plots. Sensitivity analysis was performed by the one by one elimination method of individual studies to assess the reliability of the results.

## Results

### Characteristics of the Included Studies

**Figure 1:**
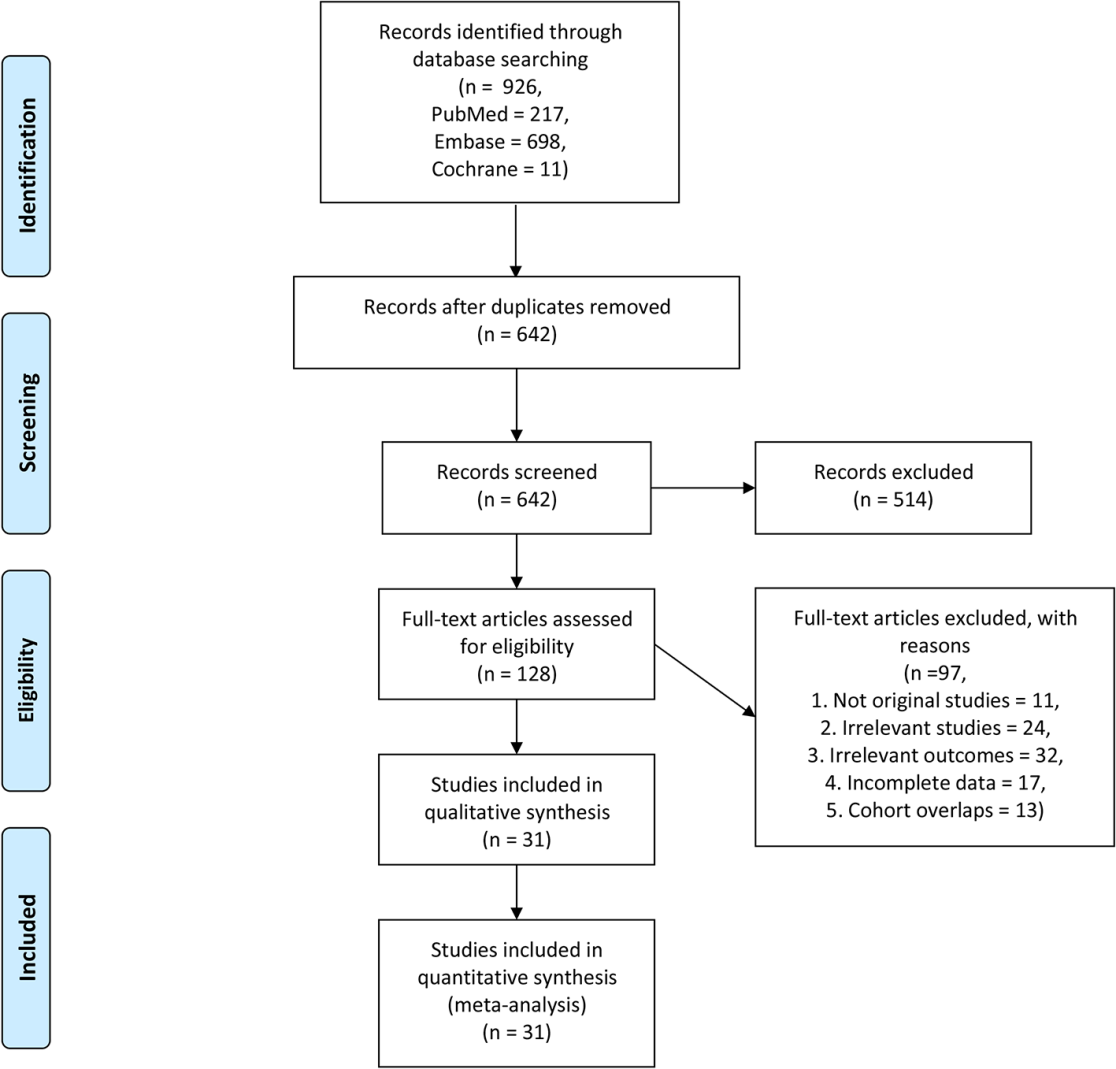
Flow chart for the study selection.

We initially identified 926 articles from the searched databases, and after excluding duplicates, 642 articles remained. After excluding 514 records, the full text of 128 articles were screened. After full-text screening, 97 studies were excluded due to the following reasons; not original studies (n = 11), irrelevant studies (n = 24), irrelevant outcomes (n = 32), incomplete data (n = 17), and the cohort overlaps (n = 13). Finally, 31 studies involving 17 tumors and a total of 3,780 patients met our inclusion criteria (**Figure 1**). The tumor types included in the study were; Nasopharyngeal Carcinoma (11), Cholangiocarcinoma (12), lung cancer (9, 10, 13–17), Hepatocellular carcinoma (20, 21, 39, 40), Thyroid carcinoma (22), Mesothelioma (23), Colorectal carcinoma (24, 25), NK/T cell lymphoma (26, 27), Peripheral T-cell lymphoma (28), Large B-Cell lymphoma (29, 35), Hodgkin lymphoma (31), Ovarian carcinoma (32), Soft tissue sarcomas (33), Renal cell carcinoma (7), Esophageal carcinoma (34), Gastric carcinoma (4, 35), and Pancreatic adenocarcinoma (36, 41).

The main characteristics of the 31 studies are summarized in **Table 1**. The NOS scores of the included studies were all greater than or equal to 6 points, except for several conference abstract articles (**Table 1**).

### Survival Analysis

**Figure 2:**
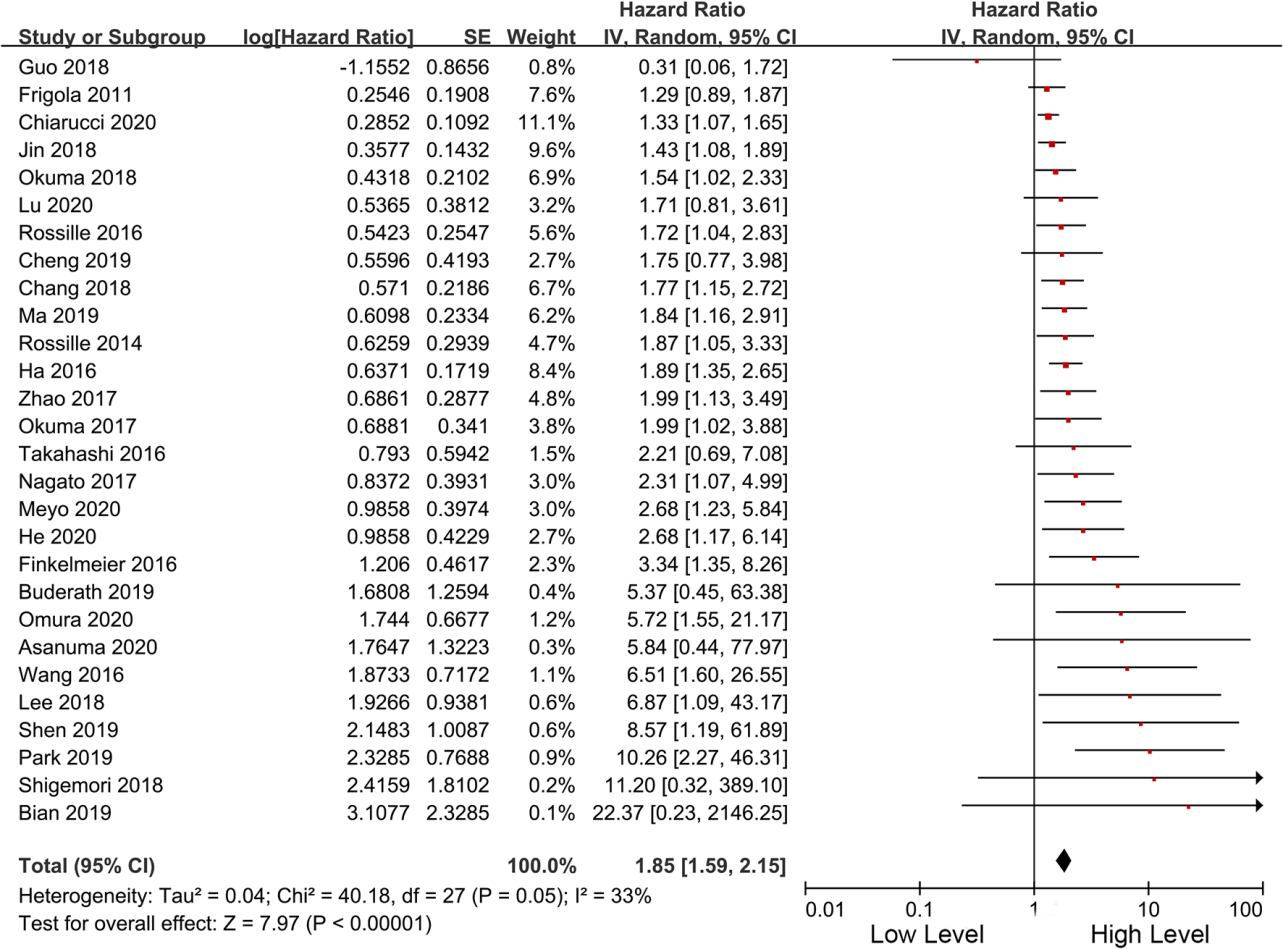
Forest plot of the overall survival in patients of high and low sPD-L1.

**Figure 3:**
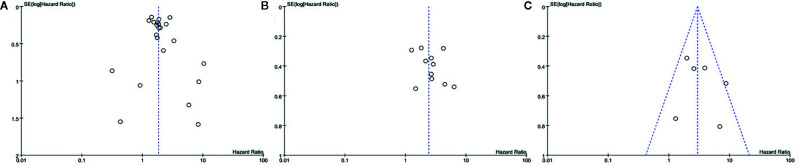
Funnel plot of the **(A)** overall survival, **(B)** PFS, and **(C)** DFS in patients of high and low sPD-L1.

Among the included studies, 28 studies have OS as their primary outcome. OS data from these 28 studies were pooled, and the results showed that overexpression of sPD-L1 was associated with shorter OS (HR 1.85, 95% CI 1.59–2.15, I^2^ = 33%) (**Figure 2**). The funnel plot showed that there was no significant publication bias (**Figure 3**). The publication bias analysis of RFS was not performed due to the limited number of included studies. Sensitivity analysis showed that the results were stable and reliable.

In terms of secondary outcomes, PFS, RFS, and DFS were also pooled for analysis. The pooled data of 11 studies showed that high sPD-L1 is correlated with worse PFS (HR 2.40, 95% CI 1.55–3.72, I^2^ = 83%). Besides, six studies included DFS data. High sPD-L1 was correlated with worse DFS (HR 2.92, 95% CI 2.02–4.29, I^2^ = 40%). Moreover, only two studies reported RFS, and sPDL1 was insignificantly correlated with a worse RFS (HR 2.08, 95% CI 0.99–4.40, I^2^ = 0%) (**Table 2**).

**Table 2:** Analyses of secondary outcomes.

Secondaryoutcomes	No. of studies	Pooled HR (95%CI)	P-value	Heterogeneity		
				Model	I^2^	P_Q_
PFS	11	2.40 (1.55–3.72)	<0.001	Random	83	<0.001
RFS	2	2.08 (0.99–4.40)	0.050	Random	0	0.670
DFS	6	2.92 (2.02–4.29)	<0.001	Random	40	0.140

CI, confidence interval; HR, hazard ratio; PFS, progression-free survival; RFS, recurrence-free survival; DFS, disease-free survival.

### Subgroup Analysis of OS

In the included 28 studies with OS data, there were different observation types, treatment methods, cut-off values of sPD-L1 and survival analysis methods. Therefore, we performed a subgroup analysis of these differences. The results showed that high sPD-L1 in both retrospective (HR 1.70, 95% CI 1.47–1.96, I^2^ = 21%) and prospective studies (HR 2.44, 95% CI 1.70–3.51, I^2^ = 32%) was correlated with worse OS. Studies were stratified according to whether surgery was performed, and the results showed that surgery did not affect the correlation between sPD-L1 and OS (surgery: HR 2.06, 95% CI 1.43–2.96, I^2^ = 31%; non-surgery: HR 1.91, 95% CI 1.57–2.32, I^2^ = 44%) (**Table 3**). The subgroup analysis based on immunotherapy revealed similar results.

**Table 3:** Results of subgroup analysis of pooled hazard ratios of OS of patients.

Stratified analysis	No. of studies	Pooled HR (95% CI)	P‐value	Heterogeneity	
				I^2^ (%)	P_Q_
Study type					
Prospective	8	2.44 (1.70–3.51)	<0.001	32	0.17
Retrospective	20	1.70 (1.47–1.96)	<0.001	21	0.19
Treatment					
Surgery	9	2.06 (1.43–2.96)	<0.001	31	0.17
Non surgery	18	1.91 (1.57–2.32)	<0.001	44	0.02
Cut-off value					
<1 ng/ml	14	2.17 (1.62–6.89)	<0.001	45	0.05
1–5 ng/ml	6	2.12 (1.42–3.17)	<0.001	44	0.11
>5ng/ml	6	1.53 (1.17–1.99)	0.002	21	0.28
Analysis					
Multivariate	22	1.83 (1.55–2.18)	<0.001	30	0.10
Univariate	6	2.07 (1.40–3.04)	<0.001	45	0.09
Immunotherapy					
No	25	1.95 (1.63–2.34)	<0.001	30	0.09
Yes	3	1.51 (1.14–1.99)	0.004	44	0.22
Detection of sPD-L1					
Pre-treatment	21	1.90 (1.56–2.32)	<0.001	38	0.04
On-treatment	3	2.19 (1.35–3.56)	0.002	22	0.28
Post-treatment	1	6.51 (1.60–26.55)	0.009	–	–

CI, confidence interval; HR, hazard ratio; OS, overall survival.

**Figure 4:**
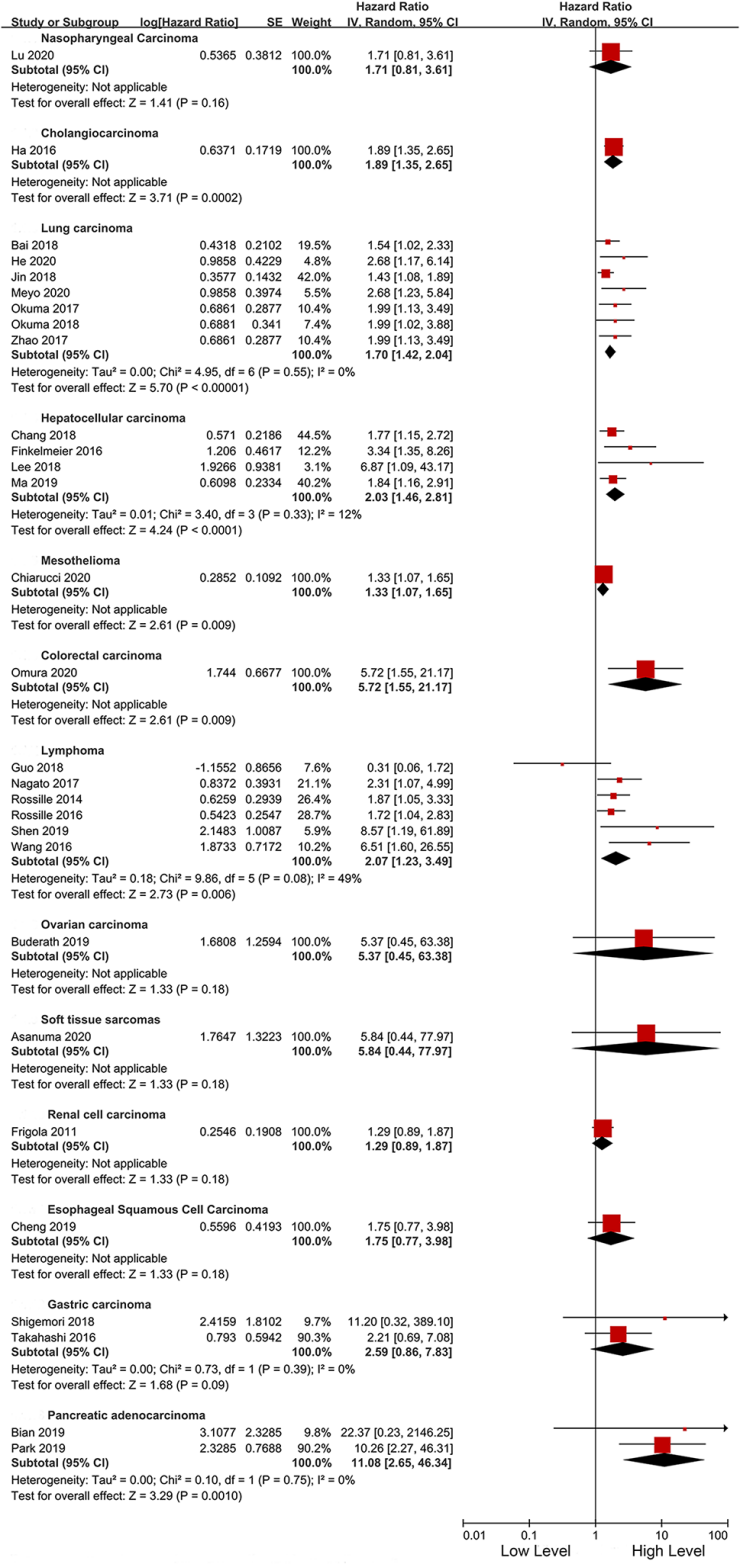
Forest plot of subgroup analysis in different types cancers.

As an important indicator, the cut-off value of sPD-L1 was divided into three levels of less than 1, 1–5, and greater than 5 ng/ml for subgroup analysis. In the three levels, high sPD-L1 was consistently correlated with worse OS (<1 ng/ml: HR 2.17, 95% CI 1.62–6.89, I2 = 45%; 1–5 ng/ml: HR 2.12, 95% CI 1.42–3.17, I2 = 44%, and >5 ng/ml: HR 1.53, 95% CI 1.17–1.99, I2 = 21%). We also performed subgroup analyses based on cut-off points (0.5 and 1 ng/ml, 1 and 10 ng/ml, and others). We found that the significant association between sPD-L1 and survival did not change with the change in cutoff points. Furthermore, sPD-L1 was associated with worse OS in both univariate (HR 2.07, 95% CI 1.40–3.04, I^2^ = 45%) and multivariate analysis (HR 1.83, 95% CI 1.55–2.18, I^2^ = 30%) (**Table 3**). We further performed subgroup analysis based on the time point of the detection of sPD-L1. Majority of the studies used the pre-treatment sPD-L1. They assessed the level of sPD-L1 at baseline when patients were initially diagnosed. The high level of sPD-L1 represented a worse OS in all three subgroups. Additionally, we performed subgroup analysis according to the specific cancer types. As presented in **Figure 4**, sPD-L1 was correlated with a worse OS in a majority of the cancers. Insignificant results were only obtained in the subgroups with less included studies (nasopharyngeal cancer, ovarian carcinoma, soft tissue sarcomas, renal cell carcinoma and esophageal carcinoma, gastric carcinoma).

## Discussion

This meta-analysis summarizes the correlation between the level of sPD-L1 and the prognosis of various cancer patients. The main significance of sPD-L1 detection is to establish a minimally invasive, easy-to-clinical, and predictive immune biomarker for cancer patients.

PD-L1 can be divided into membrane-bound PD-L1 (mPD-L1) and sPD-L1. It is believed that sPD-L1 is mainly produced by the proteolysis of mPD-L1. The immunosuppressive correlation and prognostic values of mPD-L1/sPD-L1 have not been established. Currently, IHC detection is commonly used in clinical practice to detect mPD-L1. Previous studies have explored the relationships between mPD-L1 expression levels in tumor tissues and clinical prognosis. However, these studies are limited by difficulties in sampling tumor tissues, as well as by the inability to dynamically detect changes in patients during treatment. In addition, the relationships between the levels of PD-L1 as detected by tissue IHC and the prognosis of cancer patients are not clinically binding (42, 43). Uncertainty in sampling tumor tissues, IHC staining conditions and antibody complexity, evaluator standards as well as positive cut-off values may all affect the assessment of PD-L1 levels. The advantage of sPD-L1 over mPD-L1 is that sPD-L1 can be ubiquitously present in plasma and serous effusions, and its detection is relatively convenient, repeatable and objective. A study tracked and monitored the levels of PD-L1 in plasma exosomes of patients with melanoma, and showed that all patients had exosomal-PD-L1, while only 67% of tumor biopsy specimens were PD-L1 positive (44).

We found that high sPD-L1 levels can be used as a prognostic biomarkers for poor treatment outcomes. Secondly, it has been proved that the sPD-L1 still retains its biological activity. We can further postulate that sPD-L1 in peripheral blood can be able to specifically bind T cells in peripheral blood, thereby, inhibiting T cell activity and inducing systemic immune suppression (33). When PD-L1 positive exosomes were co-cultured with activated T cells, a significant decrease in CD69 on the surface of T cells could be observed (45). The sPD-L1 can not only exert an immunosuppressive effect in the local tumor microenvironment but can also act on the distal end of the body. sPD-L1 in the blood can effectively suppress the secretion of IFN-γ by T cells, and can participate in systemic anti-tumor immune regulation by targeting T lymphocytes in secondary lymphoid organs (44, 46).

Subgroup analyses were performed based on the treatment or cut-off value of the patient. In the subgroup analysis of patients receiving immunotherapy, patients with high sPD-L1 levels were correlated with a worse OS. An NSCLC study showed that the death risk in patients with high levels of sPD-L1 was 2.68 (95%CI 1.36–5.28) times the risk in patients with low sPD-L1 levels (14). Given that sPD-L1 can be derived from tumor tissue cells, the level of sPD-L1 can reflect tumor regression to a certain extent. This implies that sPD-L1 has the potential to be used as a biomarker for predicting the prognosis of immunotherapy. Due to the high variations in sPD-L1 between various tumors and the limited number of samples, the median level of the sPD-L1 and the analyzed cut-off values are quite different. We divided sPD-L1 into three intervals for subgroup analysis, and the results consistently showed that sPD-L1 was associated with poor prognosis. The sampling error of patients and the differences between multiple tumors make it difficult for us to accurately analyze the relationship between the specific level of sPD-L1 and prognosis, however, it can still reflect the prognostic differences between patients with high and low sPD-L1. More stringent cut-off values require further exploration of large sample RCT experiments. Additionally, we also found that sPD-L1 can predict prognosis in patients subjected to, and those not subjected to surgical procedures. In NSCLC patients after radical surgery, sPD-L1 with a median value of 3.84 ng/ml could still be detected (9). This suggests that there is no release of primary foci and that there is still some detectable sPD-L1 in peripheral blood. Moreover, it implies that sPD-L1 can be derived from antigen-presenting cells in the blood. This indicates that in patients with radically resected tumors, the detection of sPD-L1 can be used to monitor immune checkpoint suppression, predict the efficacy of immunotherapy, and predict survival prognosis. This is the ability that tumor tissue IHC does not have in PD-L1 detection.

sPD-L1 can be used as a good indicator for the survival of cancer patients. Several studies have evaluated the predictive role of other immune-related biomarkers (such as soluble PD-L1, Vascular Endothelial Growth Factor A, soluble CD40L, CTLA-4, and soluble CD44) in different types of cancers (47). Meyo et al. proposed a composite biomarker using sPD-L1 and other immune-related biomarkers to predict nivolumab efficacy in NSCLC patients (13). The combination of inflammatory status indicators such as neutrophil to lymphocyte ratio with sPD-L1 has also been evaluated (12). The peripheral cytokine can be combined with sPD-L1 to predict treatment benefits and prognosis (48). The prognostic values of biomarkers that play a role in the expression of PD-L1 (such as STAT3) and their combination with sPD-L1 have also been explored (49). More combinations of sPD-L1 and other plasma indicators should be evaluated for cancer patients’ prognosis. The plasma indicators might improve the predictive performance of sPD-L1.

Two systematic reviews and meta-analysis have evaluated the relationship between sPD-L1 and tumor prognosis. The review by Ding et al. in 2017 included eight articles with a total of 1,102 cancer patients (50). Wei et al. also published a review article in 2017 that included eight articles and a total of 1,040 patients with solid tumors (51). They all found worse prognostic outcomes for patients with highly expressed sPD-L1. In recent years, a studies sPD-L1 have been published, and their results are not consistent with the above two reviews. Therefore, this review, we increased the number of studies to 31 involving 17 tumors. Compared to the previous reviews, the results of this study show that sPD-L1 is associated with worse prognostic outcomes among tumor patients receiving immunotherapy. This is different from the well-known high response rate of immunotherapy in patients with positive mPD-L1 expression (52), which also shows the different functions and significance of sPD-L1 and mPD-L1 in tumors.

This study has certain limitations. First, the studies included in this meta-analysis were observational studies, due to the lack of relevant randomized controlled trials (RCTs). Higher quality RCTs are needed to further evaluate the prognostic value of sPD-L1. Second, the number of patients with each tumor included in this study was small, and a larger sample population is needed for verification. Third, different tumor types have different molecular signatures including immune checkpoint regulators, therefore, combining them leads to inaccurate inferences and misleading clinical applications. Finally, the cut-off values of sPD-L1 in different studies were significantly different, leading to limitations in clinical applications. In future, more studies should aim at accurately establishing the correlation between sPD-L1 expression level and prognosis. However, this study shows the prognostic value of sPD-L1 in a variety of tumors and immunotherapy, indicating that sPD-L1 can potentially serve as an innovative biomarker for predicting the prognosis of cancer patients.

In conclusion, this meta-analysis revealed that high sPD-L1 levels were associated with worse survival outcomes (including OS, DFS, and PFS) in cancer patients. And among patients who had received immunotherapy, patients with high sPD-L1 levels had worse OS. The sPD-L1 may be a potential prognostic, non-invasive, and dynamic monitoring biomarker for cancers.

## Data Availability Statement

The original contributions presented in the study are included in the article/supplementary material. Further inquiries can be directed to the corresponding authors.

## Author Contributions

PH, HL: protocol/project development. PH, HL, YW: data collection and management. HL, WH: data analysis. HL, WH, YZ: manuscript writing/editing. All authors contributed to the article and approved the submitted version.

## Conflict of Interest

The authors declare that the research was conducted in the absence of any commercial or financial relationships that could be construed as a potential conflict of interest.
